# Protein kinase GIα oxidation negatively regulates antibody production by B cells

**DOI:** 10.1016/j.redox.2025.103894

**Published:** 2025-10-14

**Authors:** Hyun-Ju Cho, Rebecca L. Charles, Oleksandra Prysyazhna, Sapna Arjun, Asvi A. Francois, Kevin M. McBride, Philip Eaton

**Affiliations:** a William Harvey Research Institute, Faculty of Medicine and Dentistry, Queen Mary University of London, London, EC1M6BQ, United Kingdom; b Department of Epigenetics and Molecular Carcinogenesis, Science Park, The University of Texas MD Anderson Cancer Center, Smithville, TX, USA

## Abstract

Endogenous oxidants induce a C42-dependent interprotein disulfide between the subunits of protein kinase G (PKG) Iα in cardiovascular tissues to control blood pressure and ventricular relaxation during diastole. PKGIα is expressed in other cell types, including those of the immune system, where the redox state of this kinase is likely to regulate other important physiological processes. The role of PKGIα oxidation in antibody production by B cells, which produce oxidants such as hydrogen peroxide during differentiation, was examined by comparing the immune response of oxidation-resistant C42S PKGIα knock-in (KI) mice to their wild type (WT) littermates.

Immunization with the *4*-*hydroxy*-*3*-*nitrophenyl acetyl* (NP)-chicken gamma globulin with adjuvant increased NP + B cells (NP + B220+), germinal center B cells (NP + GL-7+CD95^+^), IgG1+ B cells (NP + IgG1+B220+), plasmablast (NP + CD138+B220+) and plasma cells (NP + CD138+B220-) in spleen, with these responses being significantly potentiated in KI mice. The C42S PKGIα KI mice also secreted significantly more NP + IgG1 antibody in plasma compared to WTs. Adoptive transfer of B cells into B cell-deficient mice confirmed that the observed enhancements were intrinsic to the donor population.

PKGIα physically interacted with activation-induced cytidine deaminase (AID), an essential enzyme for antibody diversity by deamination of cytosine to uracil in DNA, phosphorylating it at S38 to increase activity. Oxidation of PKGIα to interprotein disulfide form reduced its ability to bind and phosphorylate AID at S38, as demonstrated by *in vitro* and *ex vivo* co-immunoprecipitation, kinase assays, class-switch recombination assays, somatic hypermutation analyses, and western blotting in both *in vitro* and *in vivo* studies. In conclusion, PKGIα C42 oxidation in B cells decreases its interaction with and phosphorylation of AID, thereby reducing their antibody production.

## Graphical abstract

**Figure:**
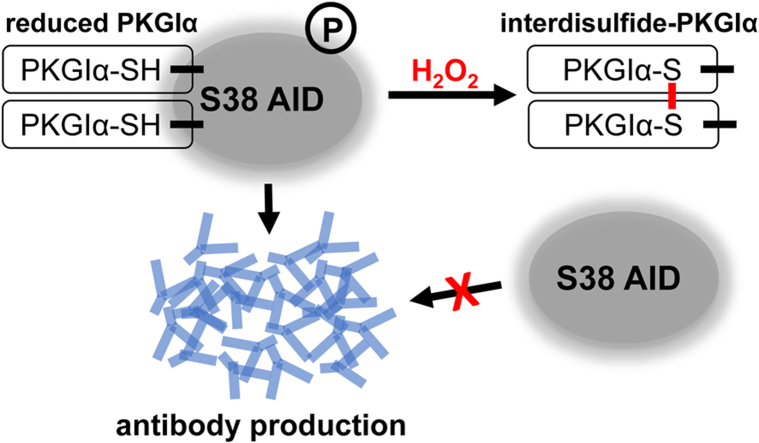

## 1 Introduction

B cells play a central role in the adaptive immune system by terminally differentiating into plasma cells that secrete antigen-specific antibody. T cell-dependent (TD) responses generate high-affinity, long-lasting antibodies, while T cell-independent (TI) responses elicit a rapid, IgM-dominated response with lower affinity [1]. Class switch recombination (CSR) and somatic hypermutation (SHM) are essential processes in the TD responses, controlling antibody diversity in germinal center B cells. CSR and SHM are initiated by activation-induced cytidine deaminase (AID), an enzyme that induces this process by introducing deamination of cytosine to uracil in single-stranded DNA [[2], [3], [4], [5]].

Protein kinase G (PKG) is classically activated by the nitric oxide (NO)-cyclic guanosine monophosphate (cGMP) pathway. Cellular oxidants induce a cysteine (C) 42-dependent interprotein disulfide between the two subunits of the PKGIα homodimer, targeting and activating the kinase to cause arterial and myocardial relaxation *in vivo* [[6], [7], [8], [9]]. This redox regulated kinase is expressed in other cell types, including B lymphocytes that mediate production of antigen-specific immunoglobulins.

Several studies have shown B cell proliferation, apoptosis and differentiation are redox regulated [10]. Indeed, B cells produce oxidants, including hydrogen peroxide that induces disulfide-PKGIα, during differentiation to plasma cells [[11], [12], [13]]. NADPDH oxidase (gp91phox subunit) knock-out mice that are deficient in superoxide and hydrogen peroxide production showed increased B cell proliferation and antigen-specific antibody production [12]. In addition, B cells that are deficient in the hydrogen peroxide generator Duox 1 proliferate faster, especially in the germinal center [14]. Inducible NO synthase (iNOS) generates large amounts of NO that enable protein S-nitrosation, a process that couples to disulfide-PKGIα formation [[15], [16], [17]]. With this in mind, it is notable that iNOS increased survival of antibody-secreting plasma cells [18]. Based on these considerations, the potential role of PKGIα redox state in the B cell immune response was investigated.

The role of disulfide-PKGIα in B cell differentiation and antibody production was explored by comparing the responses of wild type (WT) mice to a *4*-*hydroxy*-*3*-*nitrophenyl acetyl* (NP)-chicken gamma globulin (cGG) challenge with those of C42S PKGIα knock-in (KI) mice that are wholly resistant to this oxidative targeting and activation. The KI mice had potentiated responses compared to WT, generating more plasma B cells and IgG1 in response to an immune challenge with TD antigen NP. Mechanistically this was because oxidation of PKGIα decreased its binding to and the phospho-activation of AID that is integral to this immune response.

## 2 Material and methods

### 2.1 Mice and immunization

Transgenic KI mice constitutively expressing C42S PKGIα were generated by site-directed mutagenesis as previously described [6]. Mice were immunized intraperitoneally with 100 μg NP-conjugated cGG (Biosearch Technologies) combined with Alhydrogel adjuvant 2 % (InvivoGen) or 100 μg NP-conjugated dextran (Biosearch Technologies). Spleen and plasma were collected on day 7 or 14 after injection. All procedures were performed in accordance with the Home Office Guidance on the Operation of the Animals (Scientific Procedures) Act 1986 in the United Kingdom and were approved by a Queen Marry University Animal Welfare and Ethical Review Body.

### 2.2 Flow cytometry, cell sorting and sequencing

Spleen tissue was dissociated using a syringe plunger, passed through a 40 μm nylon mesh cell strainer (Thermo Fisher Scientific) and then centrifuged at 1500 rpm for 5 min. The cell pellets were resuspended in flow cytometry buffer (2 % FBS, 2 mM EDTA in PBS). Isolated single cells were pre-incubated with an Fc receptor blocker before staining with the following antibodies: Phycoerythrin (PE)-NP (Biosearch Technologies), Alexa Fluor 700 CD45R (BD), Brilliant Violet (BV) 789 CD19 (BioLegend), FITC CD21/35 (BD), PE-CF594 CD23 (BD), Alexa Fluor 647 GL7 (BD), RealBlue 780 CD95 (BD), BV 421 CD5 (BioLegend), BV510 IgG1 (BD), BV 605 CD138 (BD), BV 650 CD43 (BD), BV 711 IgM (BD), and PE-Cy7 CD93 (BioLegend). Samples were analyzed using an LSR Fortessa 2 flow cytometer (BD) and data were processed with FlowJo software (version 10.6.2, BD). Germinal center B cells were stained with Alexa Fluor 700–conjugated CD45R (BD), Alexa Fluor 647–conjugated GL7 (BD) and PE-Cy7–conjugated CD95 (BD) and then sorted using a FACSAria flow cytometer (BD). Representative FACS gating strategies are shown in Supplementary Fig. 1. DNA was extracted from sorted cells using DNeasy Blood & Tissue Kit (Qiagen) and amplified by nested PCR with Phusion Plus DNA Polymerase (Invitrogen). The variable heavy-chain region 186.2 (V 186.2) sequences were amplified using semi-nested PCR. In the external PCR, the forward primer 5′-TCTTTACAGTTACTGAGCACACAGGAC-3′ and reverse primer 5′-GGGTCTAGAGGTGTCCCTAGTCCTTCATGACC-3′ were used. For the internal PCR, the forward primer 5′-CAGTAGCAGGCTTGAGGTCTGGAC-3′ was used with the same reverse primer [19]. The PCR products were purified with a QIAquick gel extraction kit (Qiagen), cloned into a TOPO vector and transformed into competent *E. coli* cells for sequencing according to the manufacturer's instructions (Invitrogen). Plasmid DNA was sequenced with an M13 reverse primer [20]. The variable heavy-chain gene sequences were analyzed using IMGT/V-QUEST [21,22]. All analyses were performed with the IGHV1-72∗01 gene as a reference, identifying unique sequences matching the V186.2 gene. The number of mutations per sample across groups was calculated based on a sequence length of 288 base pairs.

### 2.3 NP-specific ELISA

96 well plates (Greiner bio-one) were coated with 50 μg/ml NP-7 or NP-27 BSA (Biosearch Technologies) overnight at 4 °C. The plates were washed with PBS with 0.1 % Tween-20 and blocking was with 1 % BSA for 1hr at room temperature. Serum from NP-immunized mice was diluted 1: 200 or 1:50 and 50 μl was loaded per well. The plates were incubated for 16 h at 4 °C and then washed with PBS with 0.1 % Tween-20. HRP-conjugated goat anti-mouse IgG1, IgM or IgG3 (Southern Biotech) antibodies were added. The plates were developed with tetramethylbenzidine (TMB) solution (BioLegend) and stop solution was added (Thermo Fisher Scientific). The plates were read at 450 nm (BMG Labtech) [23]. Affinity maturation was calculated as the ratio of NP7 to NP27, as both high- and low-affinity antibodies bind to NP27, whereas only high-affinity antibodies bind to NP-7 [24].

### 2.4 B cell isolation and *in vitro* class-switch recombination

Single cells were isolated from the spleen of naïve WT or KI mice. For B cells isolation, cells were incubated with anti-biotin CD43 (Ly-48) microbeads (Miltenyi Biotech) and purified using MACS LS columns according to the manufacturer's instructions. The purity of isolated B cells was checked using flow cytometry. To monitor cell division, purified B cells were labeled with 5 μM Cell Trace Violet (CTV) (Invitrogen) in PBS following the manufacturer's protocol before treatment. For stimulation, B cells were cultured in RPMI 1640 supplemented with 10 % FBS (Gibco), antibiotic-antimycotic (Gibco) and 50 μM β-mercaptoethanol and exposed to 10 μM lipopolysaccharide (LPS) (Sigma Aldrich), 20 nM IL-4 (PeproTech) or 5 μM goat anti-mouse IgM F(ab’)_2_ (Southern Biotech). On day 3, cells were stained using a LIVE/DEAD Cell Stain Kit (Invitrogen) to check their viability and BV 605 B220 and PE IgG1 (BioLegend) to assess *in vitro* class-switch recombination. Samples were analyzed using an LSR Fortessa 2 flow cytometer (BD), and data were processed with FlowJo software (version 10.6.2, BD). AID S38 phosphorylation and AID expression were measured by western immunoblotting.

### 2.5 Real-time PCR

Total RNA was extracted from immunized spleen tissues or stimulated B cells using the RNeasy Mini Kit (Qiagen) and reverse-transcribed into cDNA (Invitrogen) following the manufacturer's instructions. Each sample was run in triplicate, with 2 ng of cDNA used for SYBR-Green-based real-time PCR (Invitrogen). Reactions were performed on a QuantStudio 5 system (Thermo Fisher) according to the manufacturer's protocol. Relative gene expression was normalized to GAPDH and calculated using the ‘delta-delta Ct’ method [25]. Primers used were as follows: *Aicda* Forward: 5′-TGGAACCCTAACCTCAGCCT-3′, Reverse: 5′-TAGCCCTTCCCAGGCTTTGA-3′, *Prdm1* Forward: 5′-TCAAGCCGAGGCATCCTTAC-3′, Reverse: 5′-AGCGTGTTCCCTTCGGTATG-3′, *Gapdh* Forward: 5′-AGGTCGGTGTGAACGGATTTG-3′, Reverse: 5′-GGGGTCGTTGATGGCAACA-3’ (PrimerBank). *Iγ1-Cγ1* Forward: 5′-TCGAGAAGCCTGAGGAATGTG-3′, Reverse: 5′-GGATCCAGAGTTCCAGGTCACT-3′, *Iμ-Cγ1* Forward: 5′-CCAGGCACCGCAAATGCC-3′, Reverse: 5′-GGACAGTCACTGAGCTGCTC-3′, *Iγ3-Cγ3* Forward: 5′-GAGGTGGCCAGAGGAGCAAGAT-3′, Reverse: 5′-AGCCAGGGACCAAGGGATAGAC-3′, *Iμ-Cγ3* Forward: 5′-ACCTGGGAATGTATGGTTGTGGCTT-3′, Reverse: 5′-AGCCAGGGACCAAGGGATAGAC-3′, *Iα-Cα* Forward: 5′-CAAGAAGGAGAAGGTGATTCAG-3′, Reverse: 5′-GAGCTGGTGGGAGTGTCAGTG-3′, *Iμ-Cα* Forward: 5′-ACCTGGGAATGTATGGTTGTGGCTT-3′, Reverse: 5′-GAGCTGGTGGGAGTGTCAGTG-3’ [26].

### 2.6 Adoptive B cell transfer

A total of 2x10^7^ purified B cells from spleen of naïve WT or KI mice were intravenously transferred into muMt mice that cannot generate mature B cells (B6.129S2-Ighmtm1Cgn/J, The Jackson Laboratory). The following day, the recipient mice were immunized intraperitoneally with 100 μg NP-conjugated cGG (Biosearch Technologies) mixed with Alhydrogel adjuvant 2 % (InvivoGen). Spleen and plasma were collected on day 7 after the injection.

### 2.7 Western blot and co-immunoprecipitation

Spleen tissue lysates were assessed for protein expression using western immunoblotting by probing with antibodies to PKG (Enzo life sciences), AID S38 phosphorylation antibody (kindly provided by Dr. Kevin McBride) [3], total AID (Cell Signaling), Replication protein A (RPA) 32/2 (Cell Signaling), Uracil-DNA glycosylase (UNG) (Thermo Fisher Scientific), Blimp-1 (Cell signaling), GAPDH (Cell Signaling) or Actin (Santacruz Biotechnology). To monitor the potential interaction of AID with PKGIα, a co-immunoprecipitation (IP) experiment was performed. 200 ng of FLAG-tag AID, HIS–V5-tag WT PKGIα or C42S PKGIα plasmid were transfected into HEK cells. After 24 h, protein was extracted with lysis buffer (50 mM Tris-HCl pH 7.4, 150 mM NaCl, 10 % NP-40 and EDTA-free proteinase inhibitor cocktail (Roche). Cell lysates were immunoprecipitated with anti-FLAG/M2 agarose beads (Sigma) for 2 h or Ni-NTA (Qiagen) for 18 h at 4 °C with or without TCEP (tris (2-carboxyethyl) phosphine). Immunocaptured proteins were eluted with sample buffer and analyzed using western immunoblotting with antibodies to FLAG (Cell Signaling) or V5 (Abcam).

### 2.8 Immunofluorescence staining and proximity ligation assays (PLA) assay

Spleen tissue was collected 7 days after immunization and embedded in OCT. Sections were stained with AID (Invitrogen), Fluorescein labeled Peanut agglutinin (PNA), IgD-BIOT (Southern Biotech) and DAPI (Sigma), and visualized using an LSM 880 microscope with Airyscan Fast. To measure PKGIα and AID interactions in spleens of WT and KI mice, PLA was performed with Duolink® In Situ Red Mouse/Rabbit (Sigma) according to the manufacturer's instructions. Anti-mouse AID and anti-rabbit PKG (Enzo life sciences) diluted 1:100 and incubated over 16 h at 4 °C. Samples were analyzed with an EVOS M7000 Imaging System (Thermo Fisher Scientific) and quantified with Image J (NIH).

### 2.9 Protein purification and *in vitro* kinase assay

3 μg DNA/ml HIS–V5-tag WT PKGIα or C42S PKGIα plasmid was transfected into 3 X 10^6^/ml shaking HEK cells with 9 μg polyethylenimine, and 2.2 mM valproic acid added the following day. Transfected cells were harvested after 5 days by adding lysis buffer (50 mM Tris-HCl pH 7.4, 300 mM NaCl, EDTA free proteinase inhibitor cocktail (Roche) and 10 mM imidazole). Protein was extracted by a freeze-thaw lysis method and immunoprecipitated with Ni-NTA (Qiagen) for 1 h at 4 °C with tube rotators. Beads were washed 3 times with washing buffer (50 mM Tris pH7.4, 300 mM NaCl and 20 mM imidazole). Protein was eluted with 500 mM imidazole using centrifuge columns (Pierce) and dialysis performed using dialysis cassettes (Pierce) for at least 18 h to remove the imidazole. BCA assay was performed to measure protein concentration. To measure phosphorylation of AID by PKGIα, 600 ng WT or C42S PKGIα was incubated with or without dithiothreitol (DTT) for 30 min at 30 °C, and then 100 ng AID, 2 mM ATP, 20 mM MgCl2 was added with or without 50 μM cGMP. Samples were collected after 15 min by adding SDS sample buffer to terminate the reaction.

### 2.10 Statistical analysis

Difference between groups was calculated by a student t-test or analysis of variance followed by a Tukey *post-hoc* test. P < 0.05 was considered significantly different.

## 3 Results

### 3.1 PKGIα C42 oxidation to the disulfide inhibits IgG1 antibody production by B cells

To determine whether PKGIα oxidation affects antibody production by B cells during TD responses**,** the immune responses of oxidation-resistant C42S PKGIα KI mice that cannot form a disulfide bond at C42 were compared to WT littermates. Immunization of WT mice with NP-cGG combined with adjuvant increased disulfide-PKGIα disulfide bond formation on day 7 compared to naïve WT mice. In contrast C42S PKGIα KI did not show this immunization-induced gel shift that indicates kinase oxidation, consistent with the absence of the cysteine necessary for interprotein disulfide formation (Fig. 1A). Immunization of mice of either genotype with NP-cGG increased NP + cells (Fig. 1B), and NP + B cells (Fig. 1C) in the splenic lymphocyte gate, but this response was significantly greater in those from KIs. NP + B-1 cells and B-1a cells, linked to innate immunity, were comparable between genotypes, while NP + B-2 cells, associated with adaptive immunity, were significantly elevated in KI mice within the splenic lymphocyte gate (Fig. 1D and E). NP + transitional B cells, an intermediate stage between bone marrow immature cells and mature splenic B cells, were increased more in KI mice in the splenic lymphocyte gate (Fig. 1F). NP + T1, T2 and T3 B cells, representing sequential maturation stages, also showed a trend to increase in KI mice in the splenic lymphocyte gate (Fig. 1G). NP + follicular B cells (NP + FoB) (Fig. 1H), germinal center B cells (NP + GCB) (Fig. 1I) were significantly increased in immunized KI mice, whereas NP + marginal zone B cells (NP + MzB) (Fig. 1H) were decreased compared to WT mice. Additionally, the total number of NP + IgG1+ cells (Fig. 1J) and NP + plasma cells and plasmablast (PB) (Fig. 1K) also significantly increased in NP-immunized KI mice compared to WTs. Overall, these results indicate that KI mice promote a stronger B cell immune response to NP antigen stimulation than WT mice. Consistent with these observations, KI mice showed significantly greater secretion of high-affinity (NP7) NP-specific IgG1 in plasma together with an increased NP7/NP27 affinity ratio 7 days (Fig. 1L) and 14 days (Fig. 1M) after NP-immunization compared to WTs. NP-specific IgG3 levels and affinity ratio were significantly elevated in immunized KI mice on day 14 (Fig. 1N). In contrast, high-affinity NP-specific IgM secretion was comparable between genotypes, while the affinity ratio was markedly reduced in NP-immunized KI mice (Fig. 1O). The expression of Blimp-1, a key transcription factor involved in plasma cell differentiation, and its encoding gene *Prdm1* was examined. Following immunization, the expression levels of *Prdm1* and Blimp-1 were significantly increased in KI mice compared to WT mice (Fig. 1P). Lastly, immunoglobulin heavy-chain variable region mutations were significantly increased in KI mice after immunization compared to WT mice (Fig. 1Q).

**Fig. 1:**
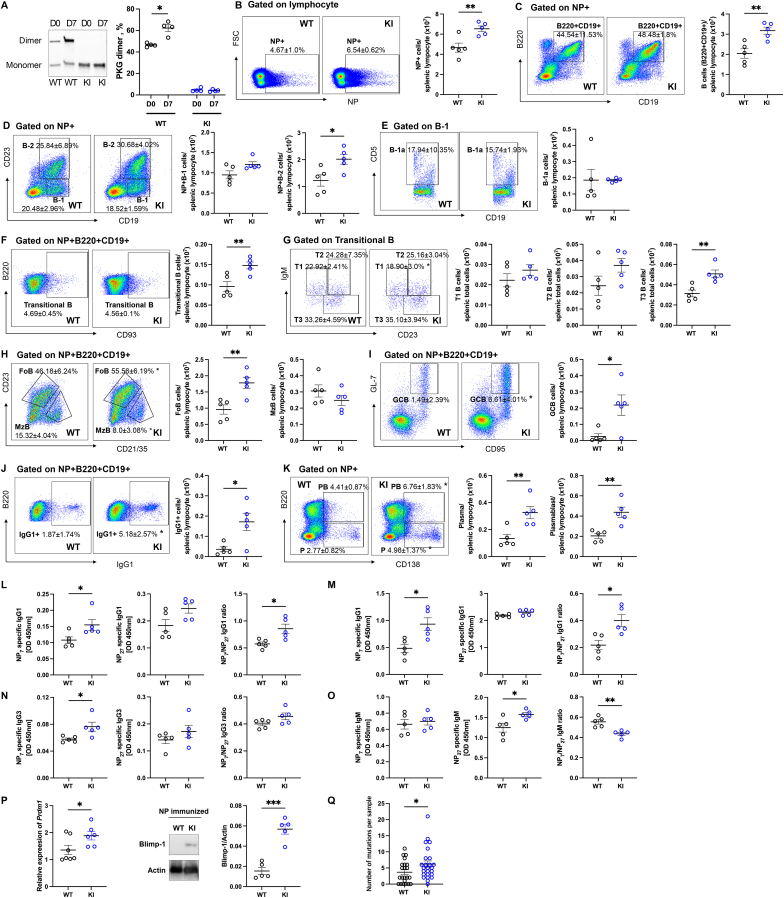
Oxidation of PKGIα negatively regulated antibody production WT or KI mice were injected with NP-cGG with adjuvant after which spleen and plasma were collected on day 7 or 14 (A) PKGIα oxidation (i.e., to the interprotein disulfide state). increased in the spleens of WT, but not oxidation-resistant KI, mice immunized with NP on day 7. Data shown as mean ± SEM (n = 4). ∗P = 0.003, unpaired two-tailed *t*-test between non-immunized and NP-immunized WT mice. (B–C) Immunized KI mice showed significantly increased NP + cells (NP + FSC+) and NP + B cells (NP + B220+CD19^+^) within the lymphocyte gate compared to immunized WT mice on day 7. (D–E) NP + B-1 cells (NP + CD19+CD23^−^) and B-1a cells (NP + CD19+CD23^−^CD5^+^) showed no significant difference between genotypes, while NP + B-2 (NP + CD19+CD23^+^) cells were significantly increased in KI mice under the lymphocyte gate. (F–G) NP + transitional B cells (NP + B220+CD19+CD93^+^) and sequential maturation stages T3 were significantly increased in KI mice within the lymphocyte gate. (H) NP + FoB cells (NP + B220+CD19+CD23+CD21/23-) were significantly increased, whereas NP + MzB cells (NP + B220+CD19+CD23^−^CD21/23+) were decreased in immunized KI mice. (I–K) NP + GCB (NP + B220+CD19+CD95+GL-7+), NP + IgG1 B cells (NP + B220+CD19+IgG1+), and NP + plasmablast (NP + B220+CD138+) and NP + plasma (NP + B220-CD138+) were significantly increased in immunized KI mice compared to immunized WT mice. The bar graphs (B–K) represent the number of various B cell subsets within the splenic lymphocyte gate. Data shown as mean ± SEM (n = 5). ∗P < 0.05, unpaired two-tailed *t*-test between NP-immunized WT and NP-immunized KI mice. (L) High-affinity NP7-specific IgG1 secretion in plasma and NP7/27 affinity ratio were significantly increased in KI mice compared to WT on day 7 after immunization. Data shown as mean ± SEM (n = 5). ∗P < 0.05, unpaired two-tailed *t*-test between NP-immunized WT and NP-immunized KI mice. (M) Similar patterns were observed NP-specific IgG1 on day 14 after immunization, (N) NP7-specific IgG3 levels and affinity ratio were also significantly elevated in KI mice on day 14. (O) In contrast, NP7-specific IgM secretion was similar between genotypes, but the affinity ratio was significantly reduced in KI mice on day 14 after immunization. (L–N) Data shown as mean ± SEM (n = 5). ∗P < 0.05, unpaired two-tailed *t*-test between NP-immunized WT and NP-immunized KI mice. (P) *Prdm1* and Blimp-1 expression were significantly increased in immunized KI mice compared with WT mice on day 7. *Prdm1* and Blimp-1 expression were measured by real-time PCR (n = 7 for NP-immunized WT and n = 6 for NP-immunized KI) and Western blot (n = 5 for each group). Data shown as mean ± SEM. ∗P < 0.05, unpaired two-tailed *t*-test between NP-immunized WT and NP-immunized KI mice. (Q) Dot plot showing the number of somatic hypermutations in the V186.2 region (288 base pairs) per sample from immunized WT and KI mice. KI mice exhibit a significantly higher frequency of SHM compared to WT mice. Data shown as mean ± SEM. ∗P < 0.05, unpaired two-tailed *t*-test between NP-immunized WT (n = 22 colonies from 8 immunized mice) and NP-immunized KI (n = 25 colonies from 9 immunized mice).

To assess the possibility that PKGIα oxidation in non-B cells modulates B cell responses, adoptive transfer experiments were performed. Purified B cells from WT or KI mice were transferred into B cell-deficient recipients, followed by NP-cGG with adjuvant immunization to determine whether the effects are B cell intrinsic. 7 days after immunization, splenic B cell subsets were analyzed by flow cytometry. B cells from WT and KI mice were successfully reconstituted into the spleens of recipient mice showing no difference in their engraftment between genotypes (Fig. 2A). However, when the B cell-deficient mice received KI B cells they showed a significant increase in antigen-specific NP + B cells (Fig. 2B), NP + GCB cells (Fig. 2C) and NP + IgG1+ cells (Fig. 2D), indicating enhanced antigen-specific activation as well as class-switching by B cells. Consistently, B cell-deficient mice that received KI B cells showed significantly increased plasma secretion of high-affinity NP-specific IgG1 and higher NP7/NP27 affinity ratio on day 7 after NP-immunization compared to WTs (Fig. 2E). In contrast, high-affinity NP-specific IgM secretion and the affinity ratio remained comparable between genotypes (Fig. 2F). The selective expansion of FoB and GCB cells (Fig. 1), supported by adoptive B cell transfer results (Fig. 2), demonstrates that the enhanced NP^+^ B cell responses and antibody production in KI mice are B cell-intrinsic and NP antigen–driven. These findings indicate that the responses reflect genuine antigen-specific B cell activity rather than baseline developmental differences, myeloid contamination, or indirect effects from other immune cells. Taken together, these data indicate PKGIα C42 oxidation to the disulfide state negatively regulated antigen-specific IgG1 production and secretion by B cells.

**Fig. 2:**
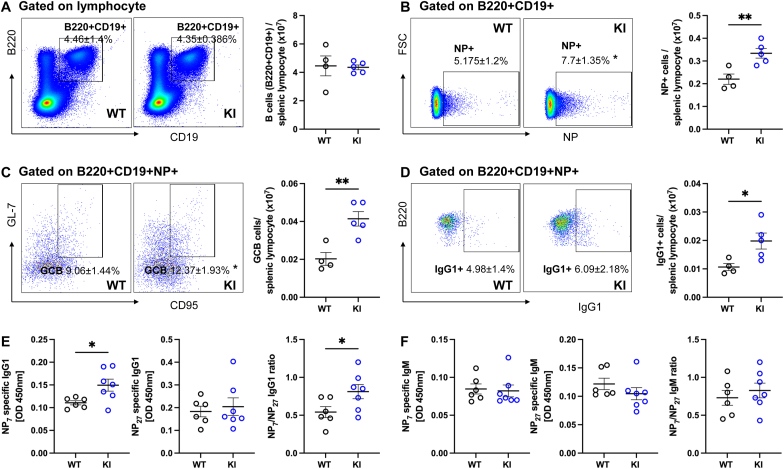
Oxidation of PKGIα attenuates B cell intrinsic regulation of antigen-specific IgG1 production Purified B cells from WT or KI mice were transferred into B cell-deficient mice, followed by NP-cGG with adjuvant immunization. Spleen and plasma were collected on day 7. (A) Engraftment efficiency of transferred B cells (B220+CD19^+^) was similar between genotypes. (B–C) Representative flow cytometry plots showed immunized KI B cell recipient mice had significantly increased in NP + B cells (B220+CD19+NP+) and NP + GCB cells (B220+CD19+NP + CD95+GL-7+) compared to immunized WT B cell recipient mice. (D) NP + IgG1+ B cells were significantly increased in immunized B cell-deficient mice that received KI B cells under the lymphocyte gate. The bar graphs (A–D) represent the number of various B cell subsets within the splenic lymphocyte gate. Data shown as mean ± SEM. ∗P < 0.05, unpaired two-tailed *t*-test between NP-immunized WT (n = 4) and NP-immunized KI (n = 5) mice. (E) High-affinity NP7-specific IgG1 secretion in plasma and NP7/27 affinity ratio were significantly increased in immunized KI B cell recipient mice compared to immunized WT B cell recipient mice on day 7 after immunization. (F) NP-specific IgM secretion in plasma was did not differ between immunized B cell-deficient mice that received either WT or KI B cells. Data shown as mean ± SEM. ∗P < 0.05, unpaired two-tailed *t*-test between NP-immunized WT (n = 6) and NP-immunized KI (n = 7) mice.

### 3.2 PKGIα physically interacts with AID

AID is important for antibody diversity through deamination of cytosine to uracil [27]. The cyclic nucleotide regulated serine/threonine kinase member protein kinase A (PKA) controls AID function by binding and phosphorylating it [28,29]. We hypothesized that PKGIα, which has similarities to PKA and is also cyclic nucleotide controlled [30], may similarly binding- and phospho-regulate AID, especially as these kinases have significant active site homology and overlapping substrate specificities [31]. Furthermore, as PKGIα activity is redox state dependent, this would potentially account for both our observation that C42S PKGIα KI mice have potentiated B cell antibody production, as well as the literature cited in the introduction showing B cells produce and are regulated by oxidants.

**Fig. 3:**
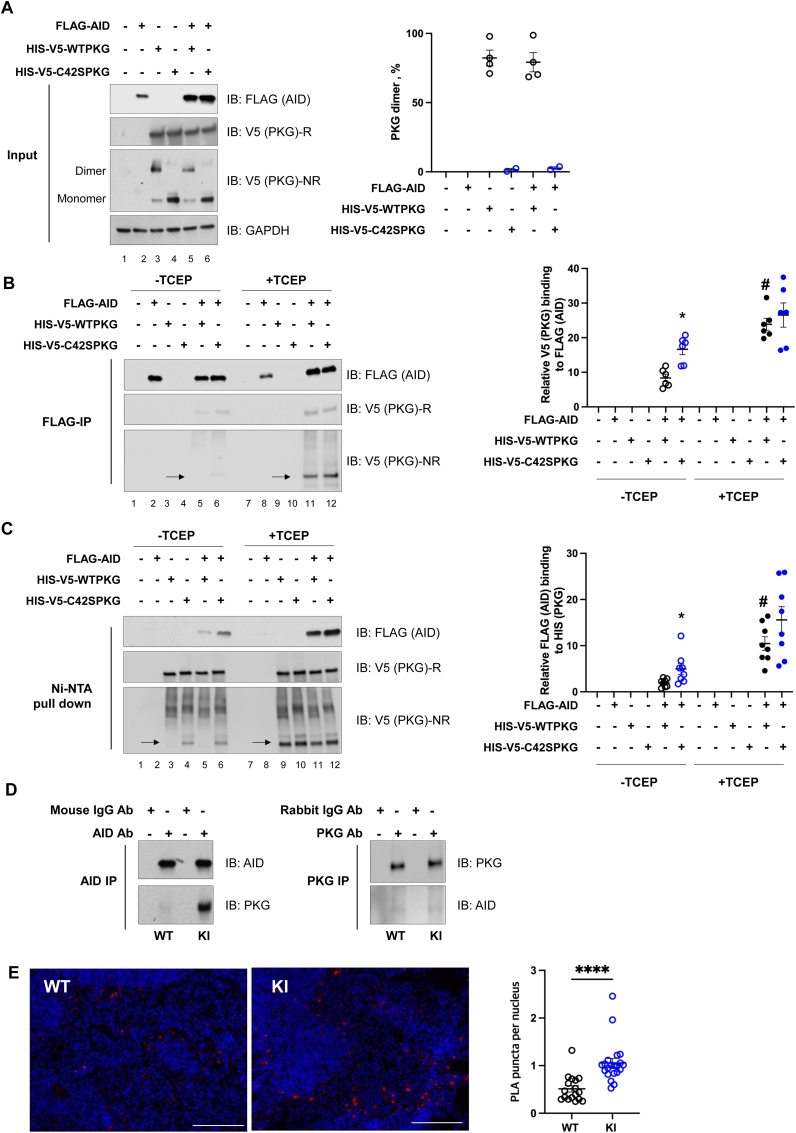
Oxidation of PKGIα to the disulfide suppressed its interaction with AID HEK cells were co-transfected with FLAG-AID, HIS–V5-WT PKGIα or C42S PKGIα plasmid and immunoprecipitated with FLAG for AID or Ni-NTA for WT PKGIα or C42S PKGIα. Soluble fraction of cell lysate (input) and immunoprecipitated samples were analyzed by Western blot under reducing (R) or non-reducing (NR) conditions and analyzed with FLAG or V5 antibodies. (A) Expression of the transfected plasmid of the cells was confirmed in input. In addition, only WT PKGIα plasmid transfected cells formed a disulfide dimer. Data shown as mean ± SEM (n = 4). (B) Immunoprecipitation of FLAG/M2 showed AID binds both WT PKGIα and C42S PKGIα. However, C42S PKGIα bound AID to a greater extent than WT PKGIα that contained a disulfide under these conditions (lanes 5 and 6). When WT PKGIα was exposed to the disulfide reductant TCEP, this increase binding with AID (lanes 11) compared to when this disulfide reductant was absent (lane 5). Data shown as mean ± SEM (n = 6). ∗P < 0.05, unpaired two-tailed *t*-test between FLAG-AID with HIS–V5-WT PKGIα and FLAG-AID with HIS–V5–C42S PKGIα. #P < 0.05, unpaired two-tailed *t*-test between FLAG-AID with HIS–V5-WT PKGIα and TCEP treated FLAG-AID with HIS–V5-WT PKGIα. (C) Similarly, Ni-NTA pull down assay showed WT PKGIα or C42S PKGIα each bind AID (lanes 5 and 6), but again the former did so less efficiently than the latter. WT PKGIα exposed to TCEP increased binding affinity (lanes 11) compared to omission of this disulfide reductant (lane 5). Quantification of the binding of PKGIα with AID, as determined by co-immunoprecipitation, is shown. V5 (PKG) binding to FLAG-IP and FLAG (AID) binding to Ni-NTA pull down assay in each lane was normalized to its input. Data shown as mean ± SEM (n = 8). ∗P < 0.05, unpaired two-tailed *t*-test between FLAG-AID with HIS–V5-WT PKGIα and FLAG-AID with HIS–V5–C42S PKGIα. #P < 0.05, unpaired two-tailed *t*-test between FLAG-AID with HIS–V5-WT PKGIα and TCEP treated FLAG-AID with HIS–V5-WT PKGIα. (D) PKGIα-AID interaction in whole spleen lysate from naïve WT and KI mice as determined using co-immunoprecipitation with an anti-AID antibody or anti-PKG antibody. The PKGIα-AID interaction was greater in the spleen of KI compared to WT mice (E) PKGIα-AID interaction was significantly increased in the NP-immunized KI mice compared to NP-immunized WT mice on day 14 as indexed using a PLA assay. Scale bars show 150 μm. Data shown as mean ± SEM (n = 17 PLA images from 3 immunized WT mice, n = 21 PLA images from 3 immunized KI mice). ∗P < 0.05, unpaired two-tailed *t*-test.

To determine whether PKGIα physically interacts with AID, HEK cells were co-transfected with FLAG-AID and HIS–V5-WT PKGIα or C42S PKGIα plasmids. Expression of the transfected plasmids was confirmed using immunoblotting, which also showed only WT PKGIα formed a disulfide dimer as anticipated (Fig. 3A). Immunoprecipitation of FLAG/M2 from co-transfected cells demonstrated AID binds both WT PKGIα and C42S PKGIα, with quantitative analysis showing this interaction was greater for the C42S PKGIα that cannot form the disulfide (lanes 5 and 6). The immunocapture of PKGIα using anti-FLAG was wholly dependent on the presence of FLAG-AID (lanes 3 and 4) (Fig. 3B), increasing confidence that the two proteins truly interact. Similarly, affinity capture of HIS–V5-WT PKGIα or HIS–V5–C42S PKGIα with Ni-NTA showed they each bind AID (lanes 5 and 6). Once again capture of AID by Ni-NTA was entirely dependent on the presence of PKGIα with a HIS tag (lanes 2) (Fig. 3C). It was notable that AID bound WT PKGIα significantly less efficiently (lanes 5) than C42S PKGIα that cannot form the disulfide (lanes 6) (Fig. 3B and C). To test whether oxidation of PKGIα to the disulfide dimer impairs kinase binding to AID, TCEP was added to reduce the disulfide and maintain the kinase in the –SH state during the capture protocol. WT PKGIα exposed to the disulfide reductant TCEP bound AID comparably to C42S PKGIα that mimics the reduced –SH state (lanes 11 and 12). However, in the absence of TCEP that allows disulfide-PKGIα to be sustained, the interaction of the kinase and AID was significantly attenuated (Fig. 3B and C). Thus, it is evident that oxidation of PKGIα to the disulfide dimer negatively regulates its binding with AID. Next, the PKGIα-AID interaction was measured in spleens from naïve WT or KI mice using co-immunoprecipitation studies. The PKGIα-AID interaction was greater in the spleen of KI compared to WT mice (Fig. 3D), further substantiating the evidence above that oxidation of the kinase limits binding with AID. This interaction was assessed further in the spleens of mice immunized with NP using PLA assay. Before performing PLA assays, AID expression in germinal center B cells was confirmed by immunofluorescence staining (Supplementary Fig. 2). Again, the PKGIα-AID interaction was significantly greater in the KI mice that cannot form a disulfide in PKGIα (Fig. 3E). Overall, these data indicate that PKGIα physically binds AID, and oxidation of this kinase to the interprotein disulfide state negatively regulated their interaction.

### 3.3 PKGIα phosphorylates AID at S38

**Fig. 4:**
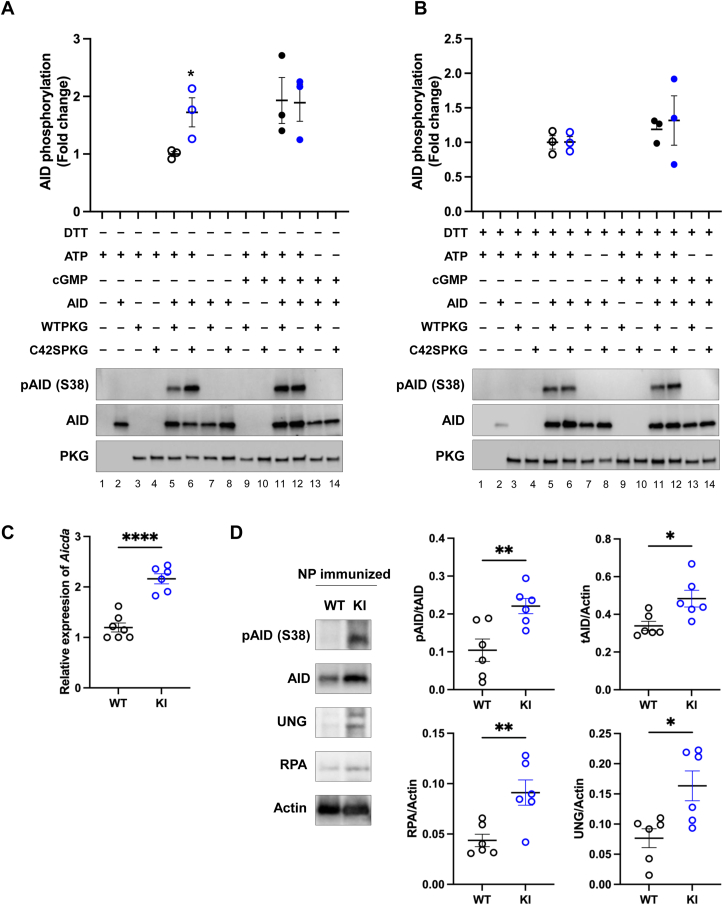
Oxidation of PKGIα impaired the AID S38 phosphorylation (A) Purified C42S PKGIα protein significantly increased phosphorylation of AID S38 as compared to purified WT PKGIα protein (lane 5 and 6). However, WT PKGIα protein treated with cGMP increased AID S38 phosphorylation as much as C42S PKGIα protein did (lane 11 and 12). Data shown as mean ± SEM (n = 3). ∗P < 0.05, unpaired two-tailed *t*-test between AID with WT PKGIα and AID with C42S PKGIα. (B) Treatment of WT PKGIα with disulfide reductant DTT increased AID S38 phosphorylation to a level equivalent to that achieved by C42S PKGIα (lane 5 and 6). However, the addition of cGMP did not further increase phosphorylation induced by the reductant DTT (lanes 11 and 12). Quantification of *in vitro* kinase assay experiments (A–B) is shown as the fold change relative to AID + WT PKGIα. AID phosphorylation in each lane was normalized to total AID. Data shown as mean ± SEM (n = 3). (C) *Aicda* transcripts were significantly increased in NP- immunized KI mice compared to WT on day 7 mice. Data shown as mean ± SEM. ∗P < 0.05, unpaired two-tailed *t*-test between NP-immunized WT (n = 7) and NP-immunized KI (n = 6) mice. (D) Representative Western blot showed spleen of KI mice immunized with NP have significantly increased AID S38 phosphorylation, total AID, RPA, and UNG expression compared to NP-immunized WT mice on day 7. Data shown as mean ± SEM (n = 6). ∗P < 0.05, unpaired two-tailed *t*-test between NP-immunized WT and NP-immunized KI mice.

Whether PKGIα phosphorylates AID and if this was altered by the oxidation state of the kinase, as anticipated from the evidence presented above showing their interaction is redox modulated, was investigated. Previous studies showed AID is phosphorylated at S38 in stimulated B cells [32,33]. Purified C42S PKGIα protein significantly increased phosphorylation of AID at S38 as determined using an *in vitro* kinase assay comparing the responses of WT or C42S PKGIα with or without the disulfide reductant DTT. WT PKGIα phosphorylated AID S38 but did so less efficiently than C42S PKGIα that mimics the reduced –SH state (lanes 5 and 6). It is notable that the reduced kinase has more activity than oxidized kinase in the absence of cGMP, and that when cGMP was added that only the oxidized form became more active (lanes 11 and 12) (Fig. 4A). When DTT was added to the reaction mixture to reduce disulfide bonds, the phosphorylation of AID by WT PKGIα showed similar phosphorylation of AID as much as C42S PKGIα did (lanes 5 and 6), reflecting the serine mutant mimicking the reduced kinase and so both forms have similar activities. Surprisingly the addition of cGMP did not further increase phosphorylation induced by the reductant DTT (lanes 11 and 12) (Fig. 4B). This unexpected finding is discussed in detail below, but likely reflects the increased interaction of reduced PKGIα with AID, whereas for other substrates enhanced interaction was observed when the kinase was oxidized [34]. *Aicda* expression (Fig. 4C) and AID S38 phosphorylation (Fig. 4D) increased in the spleens of KI compared to WT mice 7 days after NP-immunization. It was notable that this intervention also led to a concomitant increase in total AID expression**.** The AID co-factors RPA and UNG expression also increased in the spleens of KI mice, relative to WTs, 7 days after NP was administered (Fig. 4D). These data indicate that PKGIα promotes AID transcripts and phosphorylation at S38, but oxidation of this kinase to the disulfide state attenuates this.

### 3.4 PKGIα oxidation attenuates class-switch recombination

**Fig. 5:**
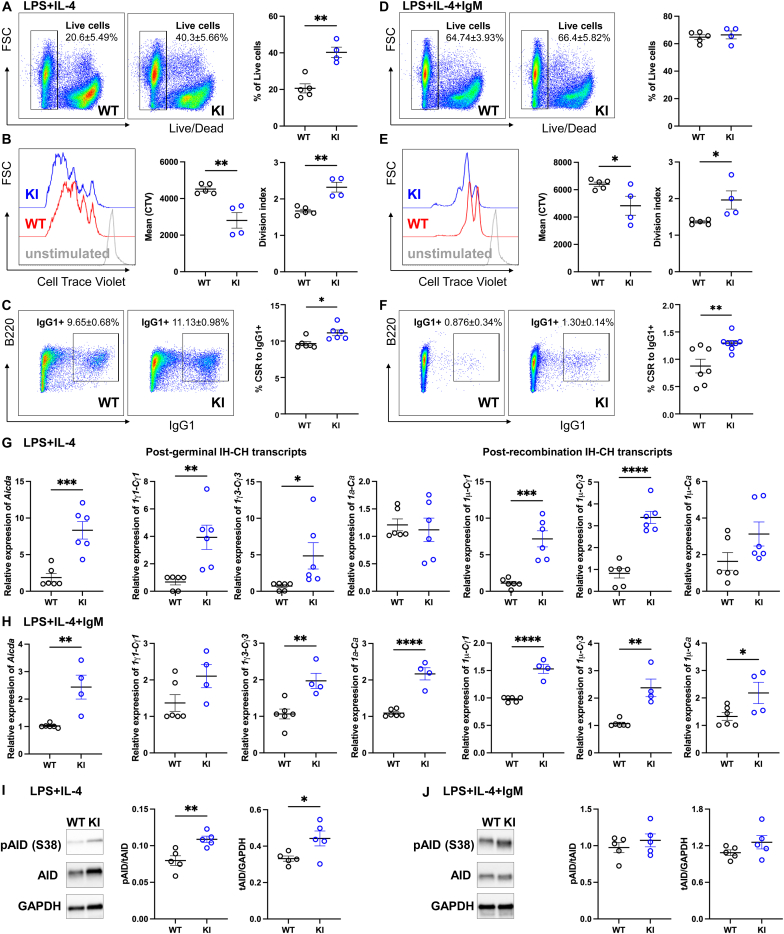
Oxidation of PKGIα attenuated cell viability, proliferation and class-switch recombination *in vitro* Primary splenic B cells from naïve WT or KI mice were stimulated with LPS + IL-4 (A-C, G and I) or LPS + IL-4 + αIgM (D-F H and J) for 3 days. (A–B) KI B cells showed significantly increased cell viability, proliferation and division compared to WT B cells under LPS + IL-4 stimulation, as measured by live/dead staining and CTV labeling, with quantification of live cells, fluorescence intensity profiles (mean) and division index analyzed by FlowJo. Data shown as mean ± SEM (WT B cells n = 5, KI B cells n = 4). ∗P < 0.05, unpaired two-tailed *t*-test. (C) In parallel experiments, IgG1+ expression was significantly greater in KI B cells than WT on day3 after LPS + IL-4 stimulation. Data shown as mean ± SEM (n = 6). ∗P < 0.05, unpaired two-tailed *t*-test. (D) No difference in cell viability was observed between genotypes under LPS + IL-4 + αIgM stimulation. Data shown as mean ± SEM (WT B cells n = 5, KI B cells n = 4). ∗P < 0.05, unpaired two-tailed *t*-test. (E) KI B cells showed significantly increased cell proliferation and division under LPS + IL-4 + αIgM stimulation. Data shown as mean ± SEM (WT B cells n = 5, KI B cells n = 4). ∗P < 0.05, unpaired two-tailed *t*-test. (F) In parallel experiments, IgG1+ expression was no difference in both genotypes after LPS + IL-4 + αIgM stimulation. Data shown as mean ± SEM (WT n = 7, KI n = 8). ∗P < 0.05, unpaired two-tailed *t*-test. (G) Under LPS + IL-4 stimulation, *Aicda, Iγ1-Cγ1, Iγ3-Cγ3, Iμ-Cγ1 and Iμ-Cγ3* transcripts were significantly increased in KI B cells compared to WT B cells, while no difference was observed in *Iα-Cα* or *Iμ-Cα* transcripts. Data shown as mean ± SEM (n = 6). ∗P < 0.05, unpaired two-tailed *t*-test. (H) Under LPS + IL-4 + αIgM stimulation, KI B cells showed significantly increased *Aicda*, *Iγ1-Cγ1, Iγ3-Cγ3, Iα-Cα, Iμ-Cγ1, Iμ-Cγ3* and *Iμ-Cα* expression. Data shown as mean ± SEM (WT B cells n = 6, KI B cells n = 4). ∗P < 0.05, unpaired two-tailed *t*-test. (I) AID S38 phosphorylation and AID expression increased significantly more in splenic B cells from KI mice compared to those from WTs on day 3 after LPS + IL-4 stimulation. Data shown as mean ± SEM (n = 5). ∗P < 0.05, unpaired two-tailed *t*-test. (J) No difference in AID S38 phosphorylation or AID expression was observed after LPS + IL-4 + αIgM stimulation. Data shown as mean ± SEM (n = 5). ∗P < 0.05, unpaired two-tailed *t*-test.

Primary splenic B cells were isolated from naïve WT or KI mice and subsequently stimulated with LPS and IL-4 with or without αIgM for 3 days to examine whether the PKGIα oxidation altered B cell viability, proliferation and activation. KI B cells showed significantly increased cell viability (Fig. 5A), proliferation and division as measured by CTV labelling (Fig. 5B), as well as increased IgG1+ labeling (Fig. 5C) compared to WT B cells following stimulation with LPS and IL-4 together. Under combined stimulation with LPS, IL-4 and αIgM, no difference in B cell viability was observed between genotypes (Fig. 5D). However, KI B cells showed greater proliferation and division compared to WT B cells (Fig. 5E). Notably, the overall classic switch to IgG1 was significantly reduced under combined LPS, IL-4 and αIgM stimulation compared to LPS and IL-4 stimulation. Nevertheless, KI B cells showed more IgG1^+^ cells (Fig. 5F). *Aicda* transcripts levels were induced in KI B cells following LPS and IL-4 stimulation (Fig. 5G) as well as stimulation with combined LPS, IL-4 and αIgM (Fig. 5H). In addition, KI B cells showed significantly increased post-germline Iγ1-Cγ1 and Iγ3-Cγ3 transcripts and post-recombination Iμ-Cγ1 and Iμ-Cγ3 transcripts following LPS and IL-4 stimulation (Fig. 5G) or LPS, IL-4 and αIgM stimulation (Fig. 5H). Iα-Cα or Iμ-Cα transcripts showed significantly difference only following LPS, IL-4 and αIgM stimulation. Furthermore, AID S38 phosphorylation and total AID expression were increased in B cells from the KI mice as compared to those from WTs under LPS and IL-4 stimulation (Fig. 5I). However, no differences were observed between genotypes following LPS, IL-4 and αIgM stimulation (Fig. 5J). Together these data are consistent with PKGIα oxidation negatively regulating immunoglobulin class-switching in B cells by attenuating AID phosphorylation by this kinase at S38.

### 3.5 PKGIα C42 oxidation impairs B cell responses induced by NP-dextran mediated immunity

**Fig. 6:**
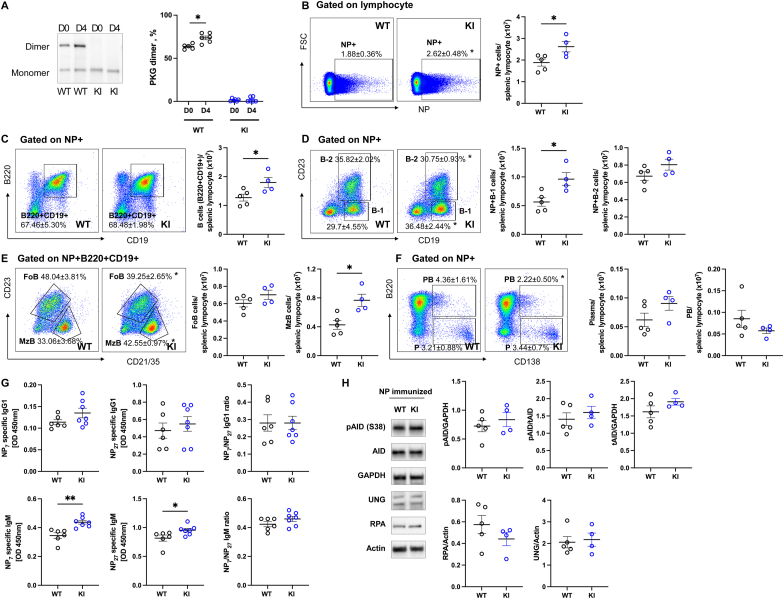
Oxidation of PKGIα impaired B cell responses to NP-dextran mediated immunity WT or KI mice were injected with NP-dextran and spleen and plasma were collected on day 4 or 7 (A) PKGIα oxidation (i.e., to the interprotein disulfide state). increased in the spleens of WT mice but not in oxidation-resistant KI mice on day 4 following immunized with NP-dextran. Data shown as mean ± SEM. ∗P = 0.005, unpaired two-tailed *t*-test between non-immunized (n = 5) and NP-immunized WT mice (n = 6). (B–C) KI mice immunized with NP-dextran showed significantly increased NP + cells (NP + FSC+) and NP + B cells (NP + B220+CD19^+^) within the lymphocyte gate compared to WT mice on day 7. (D) NP + B-1 cells (NP + CD19+CD23^−^) were significantly increased in KI mice, while NP + B-2 (NP + CD19+CD23^+^) cells were comparable between genotypes. (E) NP + FoB cells (NP + B220+CD19+CD23+CD21/23-) were similar between genotypes, while NP + MzB cells (NP + B220+CD19+CD23^−^CD21/23+) were increased in NP-dextran immunized KI mice. (F) No difference was observed in NP + plasmablast (NP + B220+CD138+) or NP + plasma (NP + B220-CD138+) between genotypes. The bar graphs (B–F) represent the number of various B cell subsets within the splenic lymphocyte gate. Data shown as mean ± SEM. ∗P < 0.05, unpaired two-tailed *t*-test between NP-immunized WT (n = 5) and NP-immunized KI (n = 4) mice. (G) NP-specific IgG1 secretion in plasma was similar between genotypes after immunization. High-affinity NP7- and low-affinity NP27-specific IgM secretion were significantly increased in KI mice, While the NP7/27 affinity ratio remained unchanged on day 7 after immunization. Data shown as mean ± SEM. ∗P < 0.05, unpaired two-tailed *t*-test between NP-immunized WT (n = 7) and NP-immunized KI (n = 9) mice. (H) No differences were observed in AID S38 phosphorylation, total AID expression, RPA and UNG expression in the spleens of immunized KI mice compared to immunized WT mice**.** Data shown as mean ± SEM. ∗P < 0.05, unpaired two-tailed *t*-test between NP-immunized WT (n = 5) and NP-immunized KI (n = 4) mice.

To investigate differences in B cell responses to TD (NP-cGG) and TI (NP-dextran) antigens, mice were immunized with NP-dextran. PKGIα disulfide bond formation was significantly increased on day 4 in WT mice immunized with NP-dextran compared to naïve WT mice. In contrast, immunized KI did not show PKGIα disulfide bond formation (Fig. 6A). Immunization KI mice significantly increased NP + cells (Fig. 6B), and NP + B cells (Fig. 6C) in the splenic lymphocyte gate. NP + B-1 cells were significantly elevated in KI mice, whereas NP + B-2 cells were comparable between genotypes (Fig. 6D). NP + MzB cells were significantly increased in immunized KI mice compared to WT mice, while NP + FoB remain unchanged in the splenic lymphocyte gate (Fig. 6E). Additionally, NP + plasma cells and plasmablast (PB) showed no difference between genotypes after immunization (Fig. 6F). Consistent with these data, KI mice showed significantly increased secretion of both high- and low-affinity NP-specific IgM, while the NP7/NP27 affinity ratio was unchanged. Additionally, IgG1 secretion in plasma showed no difference (Fig. 6G). These results indicated that NP-dextran immunized KI mice increase NP + B-1 and MzB cells in spleen and IgM secretion in plasma, consistent with PKGIα oxidation attenuating B cell responses to T cell-independent antigens. There were no changes in AID S38 phosphorylation, total AID expression, RPA and UNG expression in the spleens of immunized KI mice compared to immunized WT mice (Fig. 6H). These data indicate that T cell-independent NP-dextran stimulates the B cell immune response through an AID independent mechanism.

## 4 Discussion

This study showed that PKGIα C42 oxidation attenuated antigen-specific IgG1 production and secretion by negatively regulating its binding and phospho-activation of AID S38 during immunization with the TD antigen NP-cGG. Several studies showed that increased oxidant levels suppressed B cell proliferation [14], differentiation [12], and antibody production [11,12] and led to plasma cell apoptosis [18]. One the other hand, antioxidant interventions that may be anticipated to decrease PKGIα oxidation enhanced IgM antibody production [35]. Overall, the survival and function of B cells are influenced by their redox state, and this is at least in part mediated by modulation of PKGIα C42 oxidation.

Consistent with this, following NP-cGG immunization, AID expression was induced in both WT and C42S PKGIα KI mice. However, PKGIα oxidation via C42 dependent interprotein disulfide bond formation in WT mice significantly reduced *Aicda* transcript levels, S38 phosphorylation and protein expression compared to the oxidation-resistant transgenics. These findings indicate that disulfide-PKGIα suppresses AID induction, consistent with C42 oxidation being a key negative regulator of its expression.

PKA regulates AID function by physical interaction and phosphorylation [28,29,36,37]. PKA is a member of the serine/threonine kinase family and shares several substrates with PKGIα [31,[38], [39], [40], [41]], such as troponin I [42] and Ras-like guanine-nucleotide-binding protein (RAP1)-specific GTPase-activating proteins 2 [43]. Also, it is notable the regulatory-RIα subunit of PKA (PKARI) can be reversibly oxidized like PKGIα [41], although how this may influence B cell function appears not to have been studied. In this study we focused specifically on the role of PKG1α oxidation in B cells. We did not investigate potential synergistic effects between PKG1α and PKA or the relative contributions of each kinase. However, previous studies in cardiac and smooth muscle cell have demonstrated that PKGIα can phosphorylate and activate PKARI, and these authors discussed the prospect that oxidative stress might modulate this crosstalk [44]. Additional studies will be needed to define whether PKA interacts with PKG in regulating AID activity, particularly under conditions of oxidative stress. It will also be important to fully elucidate their relative individual contributions to regulation of AID activity and the antibody response as well as the physiological relevance of these mechanisms *in vivo*.

Here we demonstrate that PKGIα physically interacts with and phosphorylates AID, events that are enabled when C42 of the kinase is in the reduced, but not the oxidized disulfide state. Co-immunoprecipitation studies using HEK cells transfected with PKGIα and AID showed they form a complex, but kinase oxidation decreased their interaction. However, TCEP treatment, which breaks disulfide bonds, enhanced the PKGIα-AID interaction in WT PKGIα. Importantly, PKGIα and AID bound each other *in vivo* in the spleens of mice as demonstrated by co-immunoprecipitation studies, which also confirmed the interaction was greater in tissue from the KI mouse in which the kinase cannot form the disulfide at C42. Similarly, an *in vitro* kinase assay showed that phosphorylation of AID at S38 by PKGIα was significantly impaired when the kinase was oxidized, but activity was recovered when DTT was present and reduced the kinase to the –SH state. This observation could be considered surprising as oxidation of PKGIα to the disulfide increased its interaction with and phosphorylation of some substrates [34,45]. However, in contrast, oxidation of PKGIα also decreased RhoA phosphorylation [46], mirroring what was observed here with AID.

PKGIα contains several functional domains, including a leucine zipper that mediates protein-protein interactions, such as with its substrates cardiac myosin-binding protein C and myosin phosphatase [47,48]. This kinase is classically activated by cyclic GMP binding, but it can also be targeted to substrates and activated by C42 oxidation disulfide bond. As C42 is located at the proximal end of the leucine zipper, its oxidation likely influences its interactions with binding partners including substrates it phosphorylates [49]. Indeed, phosphorylation of several PKGIα substrates is regulated by the redox state of C42 [34,46,50]. What remains unclear is where PKGIα and AID bind to allow the phosphorylation of S38, such information also remains elusive for PKA that also phosphorylates the deaminase at this site [29,51]. In addition to the empirical evidence presented here showing that PKGIα binds and phosphorylates AID, this interaction could have been anticipated based on the similarity between PKA and PKG substrate consensus motifs, typically R/K–R/K-X-S/T. Notably, the AID sequence surrounding S38 (RRQS38ID) closely matches this motif [29,32,[52], [53], [54]]. Structurally, S38 resides in a solvent-exposed, flexible loop that lacks secondary structure constraints, making it accessible to kinases and likely able to adopt the conformation needed for kinase recognition. As AID does not have a leucine zipper, this raises the important question of the molecular basis of its binding to PKGIα that is rationally needed for S38 phospho-transfer. Whatever mediates this interaction and phosphorylation, it is sensitive to the redox state of C42, which is in the leucine zipper motif. However, since AID lacks a leucine zipper, we speculate that this redox sensitivity may exert its effect through an allosteric mechanism, possibly via an interacting partner or regulatory domain.

In addition to NP-cGG promoting AID phosphorylation as well as production and secretion of IgG1 specific to NP, immune stimulation increased expression of AID to a greater extent in spleens of KI mice because they lack the negative regulation enabled by disulfide-PKGIα in WT tissues. Others have reported increased AID expression upon immunes stimulation [55,56], and this likely represents a synchronized mounting of this immune response in which oxidation of PKGIα to the disulfide serves as a break. While analysis of purified GC B cells could provide additional detail, we used total splenocyte lysates based on both technical and biological considerations. GC B cells are a small fraction of splenocytes, and isolation procedures can reduce cell viability and alter PKGIα redox state. Total splenocyte lysates also capture GC-derived plasma cell activity through *Prdm1* and Blimp-1 expression, providing important information that would be missed in purified GC B cells. Thus, our approach reliably reflects GC-associated signaling and supports the conclusions drawn.

Consistent with this, RPA and UNG also showed a paralleled expression increase that was again potentiated in the KI mice. RPA is a single-strand DNA-binding protein involved in replication, recombination and repair in response to AID-mediated DNA deamination through the AID-RPA complex [36,57,58]. In addition, UNG recognizes and removes uracil generated by AID induced cytosine deamination in DNA [36,59]. These DNA repair proteins are essential for SHM and CSR together with AID [60,61]. In KI mice, NP-cGG immunization significantly upregulated the expression of AID, as well as RPA and UNG. In contrast NP-dextran, a T cell independent antigen that does not require SHM or CSR, failed to induce AID expression, and accordingly, no significant changes in RPA or UNG expression were observed between genotypes. Nonetheless, previous studies have shown that TI immunization can also induce low levels of AID expression in extrafollicular B cells or plasmablast [[62], [63], [64]]. Thus, while the AID expression was observed under TI immunization, there was no difference between the responses of WT and KI mice. These findings indicate that during immune responses such as NP-cGG immunization, AID-induced DNA damage triggers the activation of DNA repair proteins, including RPA and UNG, thereby promoting CSR and SHM. This is consistent with findings in mouse B cells showing UNG2 gene expression and enzymatic activity fluctuate in parallel with AID levels, indicating UNG2 expression is tightly coordinated with AID-mediated uracil generation [65].

These observations illustrate PKGIα activity is a significant determinant of antigen-specific IgG1 production and secretion. The finding that PKGIα phosphorylates AID at S38 to regulate IgG1 production is consistent with this deaminase being vital for CSR and SHM and thus antibody diversification. A S38A AID transgenic mouse line that cannot be phosphorylated at this residue showed significantly deficient CSR and SHM [33,66,67], highlighting a crucial role for this protein being phosphorylated by PKGIα in the redox-dependent manner as described herein.

Whether phosphorylation directly stabilizes AID protein remains unclear. USP10 specifically stabilizes nuclear AID protein and deficiency of this deubiquitinase in B cells resulted in its decreased abundance [68]. In contrast, REG-γ interacts with nuclear AID to promote its degradation, and REG-γ-deficient B cells show increased AID protein levels along with enhanced CSR [69]. There appears to be no direct evidence that phosphorylation of AID at Ser38, or phospho-mimetic mutations at this site, affects AID protein stability. Most studies have instead focused on how phosphorylation regulates AID activity [32,66]. However, phosphorylation at S38 AID promotes RPA recruitment, which may locally stabilize AID at the IgH locus and thereby enhance CSR. AID phosphorylation mutants, such as AID S38A, show significantly reduced RPA recruitment, impairing recruitment of mismatch repair proteins [66]. Consistent with this, AID^S38A/S38A^UNG ^−/−^ B cells display a block in CSR and reduced SHM [67]. In addition, the phosphatases that dephosphorylate AID S38 have yet to be identified. However, protein phosphatase 2A has been shown to dephosphorylate AID at S3, which suppresses AID activity in contrast to S38 [70]. All together, these findings suggest that while S38 phosphorylation enhances AID activity, the overall nuclear stability of AID is more directly regulated by post-translational modifiers like USP10 and REG-γ.

Previous studies have shown that the NADPH oxidase (gp91phox subunit) knock-out mice have increased B cell proliferation and enhanced antigen-specific IgG1 and IgG3 antibodies secretion in both *in vitro* CSR assays and *in vivo* TI antigen DNP-ficoll immune responses. These findings demonstrate oxidants regulate TI B cell responses [12]. Consistent with these observations, our data demonstrates that purified KI B cells showed significantly enhanced cell viability and proliferation in *in vitro* CSR experiments. Furthermore, TI antigen NP-dextran immunization in KI mice resulted in increased frequencies of NP + B-1 and MzB cells in the spleen, as well as elevated IgM secretion in plasma. Overall, these findings demonstrate oxidative signals regulate B cell responses to TI antigens, with PKGIα playing a key role in this process. Interestingly, during the *in vitro* CSR experiment, the classic switch to IgG1 was significantly reduced upon stimulation with LPS, IL-4 and αIgM together compared to that achieved by LPS and IL-4. Previous studies have indicated that αIgM treatment with LPS and IL-4 diminishes CSR without affecting cell proliferation [71]. B cell receptor cross-linking by αIgM may delay AID expression or induce phosphoinositide 3-kinase activation, potentially disrupting CSR by modulating the temporal coordination of signals necessary for this process [72,73]. This study has some limitations that should be considered. The B cell compartment in naïve mice was not examined, nor were GC size, or NP + GCB cell frequency or high-affinity antibody titers in serum at later stages such as day 21. In addition, our study is based on murine B cells and animal models, without direct validation in human B cells. Future studies using human B cells will be important to explore the potential implications for translation such as drug-induced PKGIα oxidation enhancing vaccine responses. Overall, oxidation of PKGIα to a C42-dependent interprotein disulfide abolished its binding and phospho-activation of AID, attenuating antibody production and secretion by B cells.

## CRediT authorship contribution statement

**Hyun-Ju Cho:** Writing – review & editing, Writing – original draft, Investigation, Formal analysis, Conceptualization. **Rebecca L. Charles:** Writing – review & editing, Investigation. **Oleksandra Prysyazhna:** Writing – review & editing, Investigation. **Sapna Arjun:** Writing – review & editing, Investigation. **Asvi A. Francois:** Writing – review & editing, Investigation. **Kevin M. McBride:** Writing – review & editing, Methodology. **Philip Eaton:** Writing – review & editing, Writing – original draft, Project administration, Funding acquisition, Formal analysis, Conceptualization.

## Funding

This work was supported by programme grants from the 10.13039/501100000274British Heart Foundation and the 10.13039/501100000265Medical Research Council (MR/R01065X/2), and a 10.13039/100014013UK Research and Innovation programme grant (EP/Y027698/1). R.C. is supported by a 10.13039/501100000274British Heart Foundation Intermediate Fellowship (FS/17/36/32874).

## Declaration of competing interest

"The authors declare that they have no known competing financial interests or personal relationships that could have appeared to influence the work reported in this paper."

## Figures and Tables

**Fig. 1:**
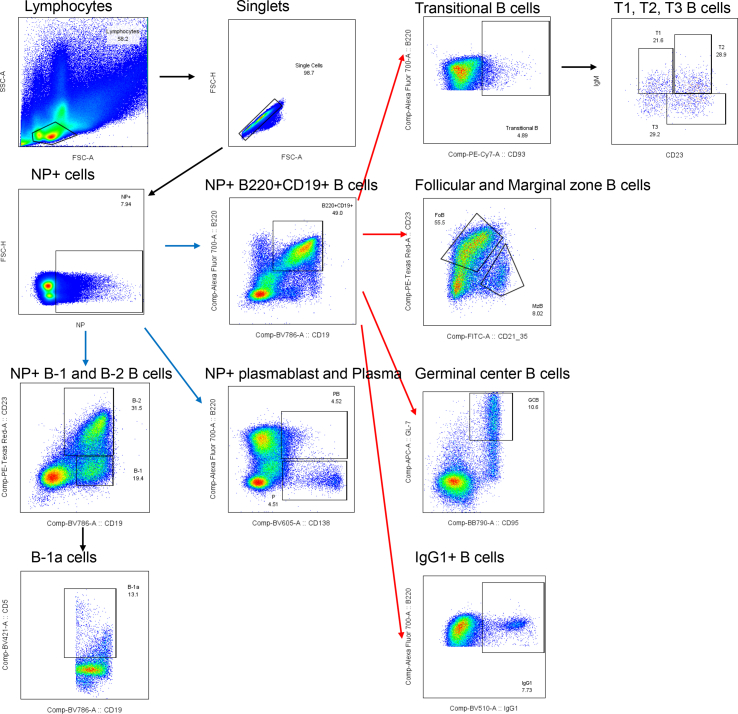
FACS gating strategy for splenic B cell subsets after *in vivo* immunization Representative FlowJo histograms showing the gating strategy used to identify various B cell subsets in the spleen of KI mice after NP-cGG immunization with adjuvant. The same gating strategy was applied to NP-dextran immunization and adoptive transfer experiments.

**Fig. 2:**
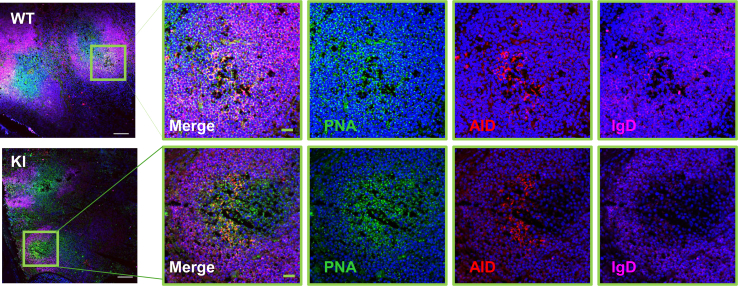
Immunofluorescence analysis of AID expression in splenic germinal center B cells Representative immunofluorescence images showed AID expression in germinal center B cells in the spleens of mice 14 days after immunization. The right panels show enlargements of the squares in the left panels (scale bar: 50 μm for low magnification, 20 μm for high magnification).

## Data Availability

Data will be made available on request.
